# Plasma biomarkers move at different rates across the Alzheimer's disease continuum

**DOI:** 10.1002/alz70856_106046

**Published:** 2026-01-08

**Authors:** Rodrigo Canovas, Christopher J Fowler, Azadeh Feizpour, Vincent Dore, Pierrick Bourgeat, Ziad S. Saad, Gallen Triana‐Baltzer, Hartmuth C. Kolb, Gwendlyn Kollmorgen, Clara Quijano Rubio, Jurgen Fripp, Simon M. Laws, Anthony W. Bannon, Kaj Blennow, Henrik Zetterberg, Stephanie R Rainey‐Smith, Christopher C. Rowe, Colin L Masters, James D. Doecke, AIBL Research Group

**Affiliations:** ^1^ Australian E‐Health Research Centre, CSIRO, Melbourne, VIC, Australia; ^2^ The Florey Institute of Neuroscience and Mental Health, The University of Melbourne, Parkville, Melbourne, VIC, Australia; ^3^ Department of Molecular Imaging & Therapy, Austin Health, Melbourne, VIC, Australia; ^4^ The Florey Institute of Neuroscience and Mental Health, Parkville, VIC, Australia; ^5^ The Australian e‐Health Research Centre, CSIRO, Brisbane, QLD, Australia; ^6^ Johnson & Johnson Innovative Medicine, San Diego, CA, USA; ^7^ Janssen Research & Development, LLC, La Jolla, CA, USA; ^8^ Enigma Biomedical Group, Knoxville, TN, USA; ^9^ Roche Diagnostics GmbH, Penzberg, Germany; ^10^ Roche Diagnostics International Ltd, Rotkreuz, Zug, Switzerland; ^11^ Centre for Precision Health, School of Medical and Health Sciences, Edith Cowan University, Joondalup, Western Australia, Australia; ^12^ Curtin Medical School, Curtin University, Bentley, Western Australia, Australia; ^13^ Collaborative Genomics and Translation Group, Edith Cowan University, Joondalup, Western Australia, Australia; ^14^ AbbVie Inc., North Chicago, IL, USA; ^15^ Department of Psychiatry and Neurochemistry, University of Gothenburg, Mölndal, Sweden; ^16^ Department of Psychiatry and Neurochemistry, Institute of Neuroscience and Physiology, the Sahlgrenska Academy, University of Gothenburg, Molndal, Sweden; ^17^ Australian Alzheimer's Research Foundation, Perth, Western Australia, Australia; ^18^ University of Western Australia, Perth, Western Australia, Australia; ^19^ Centre for Healthy Ageing, Murdoch University, Murdoch, Western Australia, Australia; ^20^ Edith Cowan University, School of Medical and Health Sciences, Centre of Excellence for Alzheimer's Disease Research & Care, Joondalup, Western Australia, Australia; ^21^ AIBL, Australia, Australia

## Abstract

**Background:**

Alzheimer's disease (AD) is a complex and multifactorial disease, defined by accumulation of Amyloid‐Beta (Aβ) and tau, and ultimately cognitive decline leading to dementia. Plasma biomarkers offer an efficient and non‐invasive method to detect either Aβ or tau pathology but have demonstrated substantially different accuracy. Assessment of how these plasma biomarkers change along the disease continuum (Aβ and tau) are needed.

**Method:**

In the current study, we investigated 15 high performing plasma biomarkers (pTau217: Lumipulse, ALZpath, Elecsys^@^ NeuroToolKit [Roche Diagnostics International Ltd, Rotkreuz, Switzerland], Janssen; pTau181: Simoa, Elecsys, UGOT; pTau231: UGOT; Aβ42/40: Lumipulse, Elecsys^@^; GFAP: Simoa, Elecsys^@^, NFL: Simoa, Elecsys, BD‐Tau: Simoa, Elecsys) against PET‐Aβ Centiloid (CL) and meta‐temporal CenTauR (CTR) across the AD continuum. Samples from the Australian Imaging, Biomarkers and Lifestyle (AIBL) study of ageing were selected if they had both plasma and PET imaging for Aβ and tau (*N* = 272). Generalised additive models from biomarker Z‐scores vs CL and CTR were computed, and receiver operating characteristic (ROC) analyses were performed to calculate area under the curve along with sensitivity, specificity, positive predictive value, negative predictive value and accuracy for both PET‐Aβ and PET‐tau positivity (CL>20 & CTR>24).

**Result:**

Against both Centiloid and CenTauR, pTau217 had the largest increases over the disease continuum, with the Z‐scores rising above the other biomarkers by 50CL for Aβ, and by 20 CTR for tau. From the point at which Aβ became positive to when tau became positive, pTau217 rose between 12% and 21%, whilst for all other markers rises of between 1% and 8% were seen. Highest correlations between biomarkers with CL and CTR were seen for pTau217 and optimal AUC values for CL and CTR reached approximate 0.95 and 0.91.

**Conclusion:**

The findings from this research confirm that pTau217 is the strongest biomarker to predict either Aβ or tau across all the four assays analyzed. Unlike other plasma biomarkers which rise with increasing Amyloid burden and stay elevated, plasma pTau217 rises with increasing Amyloid, and then again with increasing Tau burden, indicating a dual response to different AD pathologies.

